# Advancing Global Health Through Primary Care Physician Education on Suicide Prevention

**DOI:** 10.5334/aogh.4410

**Published:** 2024-05-23

**Authors:** Santiago Almanzar

**Affiliations:** 1 VA Pittsburgh Healthcare System, US

**Keywords:** Suicide, suicide prevention, mental health conditions, primary care providers, general practitioner, education, global health

## Abstract

The rising global suicide rate presents a major public health concern, resulting in the loss of over 700,000 lives annually. Discrepancies in the impact of suicide among diverse populations underscore the necessity for targeted prevention strategies. Primary care providers (PCPs) play a crucial role in identifying and managing suicide risk, particularly in underserved areas with limited access to mental health care. Educating PCPs about evidence-based interventions and suicide prevention strategies has demonstrated effectiveness in reducing suicide rates. Landmark initiatives in Australia, Sweden, and Hungary have successfully lowered suicide rates by implementing educational programs for PCPs focused on suicide prevention. Denmark, previously afflicted by some of the highest rates globally in the 1980s, has significantly reduced its figures and now ranks among countries with the lowest rates in high-income nations. Collaborative programs involving PCPs and health workers in low-resource regions have also shown promising outcomes in suicide prevention efforts. Enhancing the expertise of PCPs in suicide prevention can fortify healthcare systems, prioritize mental health, and ultimately save lives, contributing to global health endeavors aimed at addressing the pervasive issue of suicide.

## Introduction

Suicide constitutes a worldwide health concern that demands urgent attention and effective intervention strategies, with over 700,000 people taking their own lives annually, translating to a mortality rate of one person every 40 seconds [1,2]. The incidence is particularly acute among males and has witnessed a worrying uptick in the United States, where suicide rates increased approximately 36% between 2000 and 2022 [3,4]. Globally, suicide is a leading cause of death among young adults aged 15 to 29, after road injury, tuberculosis, and interpersonal violence [2]. Notably disparate suicide rates are observed worldwide, with figures soaring above 15 deaths per 100,000 in Southern Africa and Eastern Europe, while parts of Europe, South America, and Asia report fewer than 10 deaths per 100,000 annually [2].

Suicide is a widespread and complex problem that affects people from all ethnic groups, social backgrounds, religions, and regions, and can be influenced by different factors such as inherent susceptibility to mental health conditions and environmental stressors [5,6].

Disparities in suicide rates across different ages and demographics necessitate targeted prevention strategies to address the multifaceted factors contributing to suicide. Certain age groups, such as adolescents and older adults, as well as specific populations, including veterans, minorities, people in the criminal justice system, and the low- and middle-income population, are disproportionately affected by suicide, underscoring the importance of tailored support and prevention efforts. Importantly, mental health conditions such as depression, a predominant factor in suicide, often remain untreated due to systemic failures in detection and treatment.

The World Health Organization (WHO) recognizes the reduction of suicide mortality as a priority [5], with strategies including limited access to common means of suicide, responsible media reporting, the fostering of socio-emotional skills, and the management of individuals exhibiting suicidal behavior [6]. While global suicide rates have shown a decline, the issue remains critical in low- and middle-income countries (LMICs), where 77% of suicides occur, where research is sparse, and where stigma, inequality, limited access to essential health services, and a lack of mental health literacy persist [2,7,8,9,10]. Landmark initiatives in Demark have made substantial strides in decreasing suicide rates over the years, transitioning from some of the highest rates globally in the 1980s to currently boasting some of the lowest rates among high-income countries [11,12,13]. Yet, even in high-income countries, where access to mental health services and resources is relatively better, suicide prevention remains a substantial challenge, highlighting the need for more effective strategies [14,15].

There is growing evidence that suicide prevention is possible through effective treatment of psychiatric disorders and earlier detection and treatment in the general population. Psychological approaches, like cognitive behavioral therapy, show promise in preventing repeat suicide attempts. Other successful strategies include problem-solving therapies, emergency contact cards, training for general practitioners, means reduction, and public health initiatives such as reducing alcohol consumption and environmental controls such as gun policies [16,17].

The shortage of mental health care workers from various disciplines further complicates the provision of care [18]. In this environment, general practitioners can play a pivotal role in deploying prevention strategies, enhancing mental health awareness, reducing stigma, and facilitating access to care. Their role in administering interventions, such as antidepressant therapy, is crucial in the multilayered endeavor of suicide prevention. The implementation of targeted, evidence-based practices such as providing support to primary care providers (PCPs) is described as one of the most important suicide prevention strategies [16,19]. Studies have also revealed that a substantial proportion of suicide victims had been in contact with PCPs within the year leading up to their deaths, with nearly half having had contact within one month of their suicide attempt. In fact, studies have found that PCPs prescribe 79% of antidepressants and see 60% of people being treated for depression in the United States [11,16]. Given the frequency of such interactions, it is evident that PCPs are well-positioned to play a crucial role in identifying and addressing suicide risk among their patients.

This article emphasizes the critical importance of providing frontline health professionals with appropriate training in mental health and suicide prevention, especially in LMICs and rural areas. Doing so can help bridge the current gaps in mental health care, allowing for more complete, accessible, and prompt support for individuals at risk.

## Primary Care Physician Education on Suicide Prevention

Suicide prevention and mental health educational programs have demonstrated effectiveness in lowering suicide rates, both in affluent and less affluent nations. The ensuing analysis covers a spectrum of these interventions and their documented outcomes.

The PROSPECT study, a randomized controlled trial from 1999–2001 led by Bruce, Ten Have, et al., examined suicide prevention in elderly primary care patients across various US cities. Involving 598 participants with depression, randomized from aged-stratified screening, the intervention group received care based on specialized guidelines and care management. The main outcomes were suicidal ideation and depression severity, evaluated at baseline, and after 4, 8, and 12 months [20].

Key findings showed a quicker decline in suicidal thoughts among the intervention group, with a notable 12.9% drop at the four-month mark, compared to a 3.0% reduction in usual care. Suicidal ideation resolution was especially pronounced at eight months. Patients receiving the intervention displayed significant depressive symptom improvement, with the effects most marked in those with major depression unless also experiencing suicidal thoughts [20].

Despite the intervention’s success, limitations included a higher baseline suicidal ideation in the intervention group and the provision of free depression treatment to participants. Yet, two-thirds of these patients reported no suicidal ideation at four months, mirroring specialized mental health settings’ results. Broadly, the trial’s design lent to a diverse patient representation, enhancing generalizability to real-world settings. The heterogeneity in patient profiles, unique to this study, underscored the intervention’s applicability to everyday clinical practice [20].

In a separate study by Szanto, Kalmar, Hendin, and peers, a suicide prevention program was implemented in southwestern Hungary focused on General Practitioner (GP) education in depression management in Hungary, an area known for high suicide rates. Over five years, 28 GPs servicing 73,000 people were involved in an educational program accompanied by the establishment of a Depression Treatment Clinic and a psychiatrist telephone consultation service. The program’s impact was measured against a control region, the surrounding county, and national rates [21].

Primary measures included the annual suicide rate and secondary measures assessed antidepressant prescriptions. Results showed a decrease in the intervention area’s annual suicide rate from 59.7 to 49.9 per 100,000, with greater declines compared to the control area and national and county trends. Remarkably, rural female suicide rates in the intervention region declined by 34%, while a 90% increase was observed in the control region. The growth in antidepressant treatment was notably higher in the intervention region, suggesting a positive program outcome, though the authors note the need for additional measures and addressing local risk factors such as alcoholism for optimal effectiveness. This underscores the large-scale impact of primary care-based mental health interventions [21].

In the early 1980s, an educational program in Gotland, Sweden, aimed at improving GPs’ ability to diagnose and treat depression resulted in reduced sick leave and hospital admissions for depression, increased prescriptions of antidepressants, and decreased use of major tranquilizers and sedatives. Additionally, a significant decrease in suicide rates was noted. However, the positive effects of the program were short-lived and suicide rates returned to pre-program levels after it concluded, indicating that ongoing education, approximately every two years, is crucial to sustain these benefits [12].

A randomized study by Almeida and Pirkis, from 2005 to 2008 with 373 Australian GPs and 21,762 elderly patients evaluated the effect of GP education on lowering rates of depression and self-harm. GPs were randomized to receive either personalized practice audits, educational materials, and updates via newsletters, or to a control group with just general study progress newsletters without educational content. The main outcome measures were depression (Patient Health Questionnaire score ≥10) and self-harm behaviors assessed at baseline, 12 and 24 months. Results showed a 10% decline in these outcomes for patients under GPs with the educational intervention, primarily benefiting those without initial symptoms. The statistical analysis took account of the data clustering and used intention-to-treat analysis for more robust findings, demonstrating that focused educational interventions for GPs can significantly impact the mental health of older primary care patients [22].

Suicide prevention in low- and middle-income countries (LMICs) faces challenges such as limited health care access, inadequate investment in mental health, stigma, health inequities, and lack of awareness about mental health. The following program exemplify efforts to address shortages and improve access to services.

Launched in 2008, WHO’s Mental Health Gap Action Programme (mhGAP) aims to bolster mental health care and address critical issues like depression and suicide, particularly by training health care practitioners in underserved regions. In Keynejad et al., analysis of 208 studies—including 98 randomized controlled trials—mhGAP was found to significantly enhance treatment for mental disorders. Interventions such as cognitive behavioral therapy proved effective in reducing symptoms and improving patient well-being and compliance with treatments. This underscores the importance of training for GPs to strengthen the quality and reach of mental health services globally [23,24,25].

Several other projects and initiatives have been implemented globally with a focus on training GPs in countries such the UK, Australia, Chile, the US, Germany, and Japan. These initiatives have shown promising results in terms of screening rates for mental health conditions, improving the appropriate prescription of antidepressants and ultimately contributing to a reduction in suicide rates [26,27,28,29,30,31,32,33,34,35,36,37,38,39,40,41].

## Discussion

The evidence gathered and discussed in this article underscores the pivotal role that educational programs for Primary Care Physicians (PCPs) play in the detection and management of mental health conditions, ultimately contributing to suicide rate mitigation. Noteworthy outcomes from the PROSPECT study conducted in the US, along with successful models from Australia and Southwestern Hungary, indicate that strengthening GPs’ proficiency in depression care correlates with significant reductions in suicide incidences. These outcomes validate the assertion that primary care-based interventions hold substantial leverage in suicide prevention efforts.

The case study from Gotland, Sweden exemplifies the potency of educational programs in augmenting mental health services within the primary care domain. This instance also points to the necessity for continuous education in maintaining the initial improvements over time.

In low- and middle-income countries (LMICs), the uphill battle to incorporate mental health interventions and suicide prevention strategies is exacerbated by the scarcity of resources, meager mental health funding, the absence of robust mental health policy frameworks, pronounced stigma, and a widespread deficit in mental health knowledge. Such constraints pose significant impediments to effectively integrating mental health considerations into the healthcare paradigm.

Nevertheless, with interventions like the WHO’s Mental Health Gap Action Programme (mhGAP), there’s a glimmer of hope for bridging mental health gaps in resource-poor environments. By offering specialized training and crafting guidelines suited to the contexts of healthcare workers in these areas, mhGAP has evidenced appreciable strides in the enhancement of mental health care delivery and the reduction of stigmatization. The strength of the evidence, drawn from a host of randomized controlled trials, firmly attests to the utility of these educational endeavors in advancing mental health outcomes and alleviating the toll of mental ailments and suicide globally.

## Conclusion

Preventing suicide requires a variety of strategies and the dismantling of systemic barriers. Issues such as stigma, lack of awareness about mental health, limited health care services for marginalized populations, underdiagnosis, and shortage of mental health professionals are significant hurdles. These challenges make it difficult for individuals to receive the necessary care, highlighting the need to remove these obstacles.

This article proposes enhancing primary care physicians’ competence in suicide prevention through education and comprehensive training in suicide risk assessment, communication strategies, evidence-based interventions, and the integration of suicide prevention into routine clinical practice. This training should be integrated into medical school curricula, residency programs, and continuous medical education courses and standardized protocols for assessing suicide risk. Such training should aim to enhance effective communication strategies between at-risk patients and PCPs, and mental health screenings should be incorporated into routine primary care practices.

Improving the skills of PCPs and health care workers in suicide prevention is crucial to addressing this issue, especially in areas where progress has been made in screening and treatment but where wider implementation is needed. Health disparities is most pronounced in LMICs, which have relatively high rates of suicide and fewer health care resources.

## Recommendations

The author recommends enhancing suicide surveillance and community support, fighting mental health stigma through media campaigns, engaging community leaders in suicide prevention networks, increasing mental health services funding, particularly in rural areas and LMICs, and boosting research to develop targeted, region-specific interventions. These combined efforts aim to bolster prevention, create supportive environments, and ensure equitable access to mental health care, addressing suicide and mental disorders more effectively.
